# Non-manipulation of Patent LIMA in the Setting of Reoperative Aortic Valve Replacement in Patients with Previous Coronary Artery Bypass

**DOI:** 10.1186/1749-8090-10-S1-A107

**Published:** 2015-12-16

**Authors:** A Zapolanski, CE Kuschner, CK Johnson, G Ferrari, RE Shaw, ME Brizzio, JB Grau

**Affiliations:** 1 Valley Heart and Vascular Institute, Department of Cardiac Surgery, Ridgewood, NJ, USA; 2 University of Pennsylvania, Department of Surgery, Philadelphia, PA, USA

## Background/Introduction

A patent left internal mammary artery (LIMA) graft challenges the protective effects of cold blood cardioplegia in patients undergoing re-operative aortic valve replacement (AVR).

## Aims/Objectives

This study presents the results of our approach to myocardial protection in a series of consecutive patients with previous coronary artery bypass (CAB) undergoing AVR.

## Method

Between 2006 and 2014, 72 patients met criteria for inclusion. Out of these 72, 59 had previous CAB and 13 had CAB + AVR. The surgical procedures performed in these series included 50 AVR and 22 AVR + CAB. Myocardial protection was delivered using mild systemic hypothermia and continuous cold blood retrograde and intermittent antegrade cardioplegia, avoiding any manipulation of the LIMA graft. Pre-operative demographics, operative characteristics, in-hospital complications and 30-day mortality were collected according to Society of Thoracic Surgeons (STS) standards.

## Results

The cohort was predominately male (89%) and diabetic (58%) with an average age of 74 ± 8.3. Patients observed improved outcomes compared to STS predicted risk in Mortality (2.7% vs 6.5%), Stroke (1.4% vs 2.6%), Re-operation (6.8% vs 11.2%), Prolonged Ventilation (9.6% vs 21.4%), Renal Failure (1.4% vs 10.0%) Deep Sternal Wound Infection (0.0% vs 0.8%) and Length of stay >14 days (2.7% vs. 32.5%). More patients had a length of Stay >6 days than Predicted (46.6% vs 21.6%). The STS does not provide a predicted risk for myocardial infarction. The actual rates of complications were lower than expected in five of the provided categories. The 1-, 3-, 5-, and 8-year mortality rates were 4.5% (n = 3/69), 9.8% (n = 5/51), 20.0% (n = 6/30), and 45.5% (n = 5/11) respectively.

## Discussion/Conclusion

In this series avoiding the manipulation of a patent LIMA graft was associated with a low morbidity and complication rate, including myocardial infarction, when compared to the predicted STS risk. This has become our preferred approach when performing re-operative surgery on patient with patent LIMA.

